# Evaluating the reliability and validity of SF-8 with a large representative sample of urban Chinese

**DOI:** 10.1186/s12955-018-0880-4

**Published:** 2018-04-03

**Authors:** Lihua Lang, Liancheng Zhang, Ping Zhang, Qian Li, Jiang Bian, Yi Guo

**Affiliations:** 1 School of Economics, Capital University of Economics and Business, Beijing, China; 2 National Institute for Economic Experimentation, Capital University of Economics and Business, Beijing, China; 3 Institute of Economics, Chinese Academy of Social Sciences, Beijing, China; 4 Department of Health Outcomes and Biomedical Informatics, College of Medicine, University of Florida, 2004 Mowry Road, Gainesville, Florida, 32610 USA

**Keywords:** Quality of life, Health, SF-8, Reliability, Validity, Psychometrics, Urban, Chinese

## Abstract

**Background:**

The Short Form-8 (SF-8) is a widely used instrument for measuring health-related quality of life (HRQOL). The purpose of the current study is to evaluate the reliability and validity of the Chinese version SF-8 using a large, representative sample of city residents in mainland China.

**Methods:**

We surveyed residents of 35 major cities in China using random digit dialing of both landlines and cell phones. We adopted a multi-stage stratified sampling scheme and selected a probability sample of 10,885 adults. Internal consistency reliability of the SF-8 was evaluated with item-total correlations and Cronbach’s alphas. Construct validity was assessed with factor analysis. Known-groups validity was examined based on known HRQOL differences in age, gender, income, and overall quality of life.

**Results:**

We showed that SF-8 has very good internal consistency reliability and known-groups validity. Our results also confirmed that the traditional 2-factor structure of SF-8 (physical and mental health) is reasonable among Chinese city residents. Further, we showed that a 3-factor model (physical, mental, and overall health) fit the data better than the traditional 2-factor model.

**Conclusions:**

This study is the first to confirm the traditional 2-factor structure of SF-8 using a large, representative sample from China. We have shown that the SF-8 Chinese version is feasible, reliable, and valid. Our findings support the use of the SF-8 summary scores for assessing general HRQOL among Chinese. Future studies may further explore the possibility of a 3-factor structure for the SF-8 among the Chinese population.

## Background

There has been an increasing interest in measuring and assessing health-related quality of life (HRQOL) in clinical and health services research in the past a few decades [1]. Many clinical trials have included HRQOL as one of the primary trial outcomes, in addition to clinical outcomes [2]. To measure HRQOL, the Short Form-36 (SF-36) Health Survey, developed in the Medical Outcomes Study, is the most popular instrument [3]. SF-36 measures generic HRQOL among adults with 36 questions that belong to 8 sub-scales (Physical Functioning, Role Physical, Bodily Pain, General Health, Vitality, Social Functioning, Role Emotional, Mental Health), which are then used to calculate 2 summary measure scores (physical component score PCS and mental component score MCS). It has been translated into more than 170 languages and extensively tested across many different countries [4]. However, despite its popularity, the SF-36 is quite lengthy which limits its use. With 36 questions, it takes on average 17 min to complete the SF-36 survey when administered via telephone by experienced interviewers [5]. Measuring HRQOL with the SF-36 in a study would greatly increase the length of any survey, and hence the burden of study participants. Therefore, the SF-8, a shortened version of SF-36 and the shortest SF Health Survey, is preferred by many researchers [6]. With only 8 questions, the SF-8 is derived from the SF-36 for the purpose of minimizing respondent burden. While it is substantially shorter than the SF-36, administrating the SF-8 yields comparable scores for the 8 sub-scales and 2 summary measures as the SF-36. The brevity of SF-8 has made it an ideal tool to assess HRQOL, especially in large-scale observational studies where survey administration time and respondent burden are important considerations.

The SF-8 has been translated into many languages, including Spanish [7], German [8], Japanese [9], Luo [10], Korean [11], and Mandarin Chinese [12]. In a previous study, Wang et al. translated the SF-8 into Chinese following the standard International Quality of Life Assessment (IQOLA) protocol, which included forward translation, back-translation, expert review, and psychometric testing [13]. Using a random sample of 1517 participants, the authors showed that the SF-8 Chinese version has good internal consistency reliability (overall Cronbach’s alpha = 0.749) and criterion validity (correlation between the SF-8 and SF-36 was 0.559) [12]. However, the authors did not examine whether or not the 2-factor structure (physical and mental health) identified in the US is appropriate in the Chinese population. After all, the validity of PCS and MCS summary scores depends on the appropriateness of the underlying 2-factor structure. Further, the study utilized a relatively small sample of residents from a single city in Mainland China, which is not representative of the entire Chinese population. To our knowledge, Wang et al. is the only study available that has evaluated the psychometric properties of the SF-8 in China. Thus, in the current study, we extend the psychometric testing of the Chinese version SF-8 by assessing its internal consistency reliability, construct validity, and known-groups validity using a larger, more representative sample of Chinese city residents.

## Methods

### Data collection and participants

Data used in this study were collected in the 2017 Chinese City Quality of Life Survey. The goal of the Survey was to evaluate the quality of life, including HRQOL, of city residents in mainland China. Between March and May 2017, we performed random digit dialing (RDD) to interview adults aged 20 years or older residing in all major cities in China. Considering the increasing usage of mobile phones, the RDD was conducted among both landlines and mobile phones. We chose to conduct telephone surveys because: 1) face-to-face surveys were not practical given that we wished to survey a large number of participants; and 2) online surveys were often of poor data quality and population representativeness [14]. Our surveys covered all 26 provincial capitals, all 4 municipalities (Beijing, Tianjin, Shanghai, and Chongqing), and all of the 5 cities under separate state planning (Dalian, Qingdao, Ningbo, Xiamen, and Shenzhen). We adopted a multi-stage stratified sampling scheme and selected a probability sample using cities and districts within cities as strata. Computer-assisted telephone interviews (CATI) were conducted by CATI-trained students working in the survey center at Capital University of Economics and Business. The survey response rate was 18.1% and the average survey completion time was 5.2 min. The final sample included 10,885 individuals.

### The SF-8 Chinese version

**Fig. 1:**
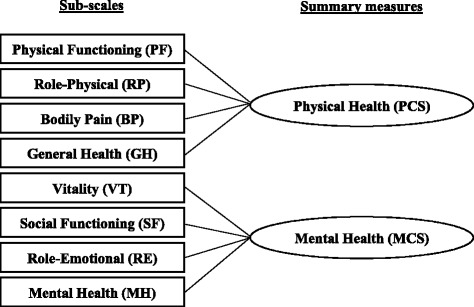
Domains of the SF-8

The SF-8 was translated into Chinese following the standard IQOLA protocol in a previous study [13]. The 8 items in SF-8 measure 8 sub-scales: physical functioning (PF), role limitations due to physical health problems (RP), bodily pain (BP), general health perceptions (GH), vitality (VT), social functioning (SF), role limitations due to emotional problems (RE), and mental health (MH) (Fig. 1). The sub-scale scores can be represented as T-scores (mean = 50; standard deviation = 10) that range from 0 to 100, with higher scores indicating better health. Two summary scores, PCS and MCS, can also be computed from the sub-scale scores. The summary scores are calculated as the weighted sum of the sub-scale scores, transformed into T-scores, and normalized to a U.S. general population. This standard-based scoring allows comparisons among SF-8 scores from different studies since these scores are normalized to the same reference population [15].

### Survey items

Besides the SF-8, we collected information on age, gender, education, income, and overall quality of life (QOL) in the surveys. Education was measured with the question “What is your education level?”. The response options were: “No formal education”, “Elementary school education”, “Middle or high school education”, “Some college or college graduate”, and “Higher than college”. Primary education (referred to as “elementary school”) in China is normally 6 years. A typical student graduates elementary school at the age of 12. Secondary education in China includes 3-year junior (referred to as “middle school”) and 3-year senior (referred to as “high school”) secondary education. A typical student graduates high school at the age of 18. Higher education (college) in China is normally 4 years. Income was measured with the question: “What is your monthly income range? (Unit: Yuan)”. The responses were “Below 2000”, “2000–4999”, “5000–7999”, “8000–15,000”, and “more than 15000”. Lastly, overall QOL was measured with the question “How would you rate your overall quality of life?”. The participants were instructed to select a number between 1 and 10, with higher numbers indicating better quality of life. All of the above questions were asked before the SF-8 in the telephone surveys.

### Statistical and psychometric analysis

We calculated frequencies and percentages to describe the demographic characteristics of our study participants. We also calculated the mean, standard deviation, and percentages of participants with the lowest (floor effect) and the highest (ceiling effect) possible scores for each of the SF-8 items. For psychometric testing, we evaluated the internal consistency reliability, construct validity, and known-groups validity of the SF-8. Internal consistency reliability was evaluated by examining the item-total correlations and the Cronbach’s alpha coefficients. The item-total correlations were calculated by removing each of the 8 items from the instrument and correlating it with the remaining items. The Cronbach’s alpha was reported for the overall instrument. We also calculated and reported the alphas when any one of the items was removed from the instrument. For Cronbach’s alpha, we considered the following cut-off values: > 0.7 (acceptable), > 0.8 (good), and >  0.9 (excellent) [16]. For item-total correlation, we considered a value greater than 0.3 to be an indicator that an item was related to the overall scale [17].

Next, we assessed construct validity with factor analysis based on a split sample approach in which the original data were randomly split into two equal halves. We performed exploratory factor analysis (EFA) with half of the sample data to explore the underlying structure of SF-8. Then, confirmatory factor analysis (CFA) was performed with the other half of the data to verify the identified factor structure. In the EFA, the factors were extracted using the principal components method with varimax rotation. The rotated factor pattern was reported and an item was considered to load on a factor if the factor loading was equal to or greater than 0.6. The number of factors extracted was determined by reviewing the scree plot and considering the following criteria: eigenvalues (> 1), proportion of total variance explained by a single factor (> 10%), and proportion of total variance explained by extracted factors combined (> 70%). In the CFA, we verified structure(s) identified in the EFA, as well as the widely accepted 2-factor (physical and mental health) structure of the SF-8. The CFA is a multivariate statistical technique used to verify whether the observed variables represent the hypothesized latent constructs. Due to the high sensitivity of the chi-square statistic in large samples [18], goodness of fit was evaluated based on the following fit indices: Standardized Root Mean Square Residual (SRMR), Root Mean Square Error of Approximation (RMSEA), and Comparative Fit Index (CFI). For SRMR, a value less than 0.05 indicates good fit [19]. For RMSEA, a value less than 0.05 indicates good fit, a value in the range of 0.05 to 0.10 indicates fair fit, and a value above 0.10 indicates poor fit [20]. For CFI and NFI, a value equal to or greater than 0.9 indicates good fit [21, 22].

Lastly, we evaluated known-groups validity to examine the extent to which SF-8 domain and summary scores can discriminate between known groups. These known groups were based on age groups, gender, education, income, and overall QOL. The QOL known groups were defined as high, median, and low overall QOL based on tertiles. We hypothesized that the SF-8 scale scores would be lower in participants who were older, women, less educated, of lower income, or of lower overall QOL [23, 24]. Standardized differences or effect sizes (ES) in SF-8 domain scores between the known groups were evaluated using Cohen’s d [25]. Based on Cohen’s suggestion, we considered 0.2 ≤ d <  0.5 a ‘“small” effect size, 0.5 ≤ d <  0.8 a “medium” effect size, and d ≥ 0.8 a “large” effect size [25]. All data analyses were performed using SAS version 9.4 (SAS Institute, Cary, NC).

## Results

### Participant characteristics

We summarized the participants’ characteristics in Table 1. The majority of the participants were between 20 and 50 years old. There was a slightly higher proportion of male participants (52.5%) than female participants (47.5%) in our sample. Regarding education, 28.9% of the participants attended or graduated high school. A little over half (55%) of the participants had some college education or a college degree. Regarding income, 56.7% of the participants reported a monthly income lower than 5000 Chinese Yuan (CNY), and 4.3% of the participants had a monthly income greater than 15,000 CNY. The mean (T-scores), standard deviation, and floor and ceiling effects of the SF-8 sub-scale scores were summarized in Table 2. The scores ranged from 51.0–57.5. The floor effects were very small for all sub-scale scores. The ceiling effects were relatively large, with the highest percentage being 72.4% for RP.

**Table 1:** Participants’ characteristics

	*n* (or mean)	% (or SD)
Age (years)		
20–30	4667	42.9%
31–40	3453	31.7%
41–50	1766	16.2%
51–60	614	5.6%
60+	385	3.5%
Gender		
Men	5714	52.5%
Women	5171	47.5%
Education		
None	300	2.8%
Elementary school	799	7.3%
Some HS or HS graduate	3144	28.9%
Some college or college graduate	5989	55.0%
Graduate school or more	653	6.0%
Monthly income		
< 2000 CNY	2884	26.5%
2000–4999 CNY	3291	30.2%
5000–7999 CNY	3093	28.4%
8000–15,000 CNY	1152	10.6%
> 15,000 CNY	465	4.3%

Note: CNY = Chinese Yuan

**Table 2:** Summary of SF-8 items

	Mean^1^	SD	% Floor	% Ceiling	Cronbach’s Alpha^2^	Item-total correlation
PF	51.0	5.3	0.2	67.3	0.81	0.68
RP	51.0	5.6	0.3	72.4	0.81	0.68
BP	56.8	6.4	0.3	66.0	0.82	0.64
GH	52.1	7.2	0.7	32.3	0.84	0.51
VT	53.9	9.2	3.9	38.5	0.85	0.39
SF	51.8	5.9	0.4	64.2	0.82	0.63
RE	49.2	5.5	0.3	68.1	0.83	0.58
MH	51.1	7.1	0.2	51.5	0.83	0.54

Note: ^1^T-scores. ^2^Cronbach’s alpha if the item is removed; cut-off values: > 0.7 (acceptable), > 0.8 (good), and > 0.9 (excellent). ^3^Greater than 0.3 indicates acceptable

### Internal consistency reliability

The overall Cronbach’s alpha was 0.85 for the 8 items. The Cronbach’s alphas calculated by removing the items from SF-8 one by one were summarized in Table 2. As seen in the table, the alpha exceeded 0.8 when any one of the items was removed (range = 0.81–0.85), indicating no single item is redundant or lowering the scale’s internal consistency. The item-total correlations for the 8 items were also summarized in Table 2. The correlations were moderate or high (*r* >  0.5) for all the items, except for VT (*r* = 0.39). Overall, the SF-8 showed very good internal consistency.

### Construct validity

We summarized results from the EFA in Table 3. The scree plot analysis identified three factors, which explained a combined 72.7% of the total variance. The proportions of the variances explained were 49.4%, 11.9%, and 11.4%, and the eigenvalues were 3.9, 1.0, and 1.0 for the 3 factors, respectively. The first factor included PF, RP, BP and GH, the items pertaining to physical health. The second factor included SF, RE, and MH, the items pertaining to mental health. The third factor included GH and VT, the items pertaining to overall health.

**Table 3:** Factor loadings from exploratory factor analysis

Items	Factor 1	Factor 2	Factor 3
PF	*0.86*	0.24	0.12
RP	*0.85*	0.30	0.05
BP	*0.67*	0.31	0.28
GH	0.52	0.06	*0.60*
VT	0.06	0.19	*0.89*
SF	0.35	*0.72*	0.14
RE	0.35	*0.77*	−0.02
MH	0.09	*0.81*	0.27

Note: significant factor loadings are italicized

Further, we conducted CFA to verify 3 models. First, we performed a 1-factor CFA in which a single latent construct was considered due to the high association between physical and mental health. Second, we performed a 2-factor CFA using the traditional factor structures which included PCS (GH, PF, RP, and BP) and MCS (VT, SF, MH, and RE) domains. Third, we performed CFA on the 3-factor model we identified. Model fit indices were summarized in Table 4. The data did not fit the 1-factor model well (SRMR = 0.07; RMSEA = 0.14; CFI = 0.87; NFI = 0.87). However, the 2-factor model exhibited a satisfactory model fit (SRMR = 0.05; RMSEA = 0.10; CFI = 0.94; NFI = 0.94). Out of the 3 models, the 3-factor model had the best model fit (SRMR = 0.04; RMSEA = 0.09; CFI = 0.94; NFI = 0.96). The AIC values decreased across the 1-, 2-, and 3-factor models, indicating that the 3-factor model had the best fit out of the 3 models.

**Table 4:** Fit indices from confirmatory factor analysis

	1-factor model	2-factor model	3-factor model
Chi-square (df)	4127.9 (19)	1887.5 (17)	1291.4 (13)
AIC	4161.99	1925.46	1337.45
SRMR	0.06	0.05	0.04
RMSEA	0.14	0.10	0.095
CFI	0.87	0.94	0.96
NFI	0.87	0.94	0.96

Notes: AIC = Akaike Information Criterion; SRMR = Standardized Root Mean Square Residual (good fit: < 0.05); RMSEA = Root Mean Square Error of Approximation (good fit: < 0.05; fair fit: 0.05–0.10); CFI = Comparative Fit Index (good fit: ≥ 0.9); NFI = Normative Fit Index (good fit: ≥ 0.9)

### Known-groups validity

We summarized results from the known-groups validity analysis in Table 5. There was an expected downward trend of physical health across the age groups. Using participants older than 60 as the reference group, the ESs for the physical health domains PF RP, and BP decreased in a consistent fashion, going from “medium” (ES: 0.54–0.60) for the 20–30 age group to “small” (ES: 0.21–0.25) for the 50–60 age group. For the mental health domains SF, RE, and MH, the ESs were mostly negligible except for the 20–30 age group for SF (ES = 0.24) and RE (ES = 0.31). Regarding gender, we did not observe any significant differences in sub-scale scores between women and men (all ESs <  0.2). Compared to participants with high school or lower education, those with more than high school education had better physical health (PF, RP, and BP) and RE scores, although the ESs were “small” (ES: 0.20–0.27). We did not observe any significant differences in sub-scale scores between the income groups (all ESs <  0.1). For the overall QOL known groups, all the domain scores increased with the overall QOL, which was consistent with prior findings.

**Table 5:** Known-groups Validity of SF-8 Based on Cohen’s d

	Age				Gender	Education	Income (CNY)	Overall QOL	
	20–30	30–40	40–50	50–60	Men	>HS	> 5000	2nd	3rd
	vs. > 60	vs. > 60	vs. > 60	vs. > 60	vs. Women	vs. ≤HS	vs. ≤5000	vs. 1st	vs. 1st
PF	0.54	0.31	0.30	0.22	0.02	0.22	0.05	0.28	0.41
RP	0.53	0.25	0.27	0.21	0.01	0.27	0.04	0.30	0.41
BP	0.60	0.39	0.31	0.25	0.04	0.22	0.06	0.30	0.46
GH	0.49	0.39	0.28	0.28	0.07	0.12	0.08	0.37	0.55
VT	0.27	0.28	0.18	0.28	0.14	0.02	0.09	0.29	0.44
SF	0.24	0.12	0.11	0.11	0.04	0.13	0.04	0.29	0.41
RE	0.31	0.13	0.10	0.15	0.03	0.20	0.06	0.32	0.40
MH	−0.06	−0.09	−0.16	−0.06	0.10	0.02	0.01	0.25	0.45

CNY: Chinese Yuan; QOL: Quality of Life; HS: High School

## Discussion

In this study, we examined the internal consistency reliability, construct validity, and known-groups validity of the Mandarin Chinese version SF-8 among city residents in mainland China. We show that SF-8 has very good internal consistency and known-groups validity. In addition, our results indicate that the traditional 2-factor structure of SF-8 (physical and mental health) is reasonable among Chinese city residents. Further, our results show that a 3-factor model (physical, mental, and overall health) fits the data better than the traditional 2-factor model.

The study participants responded consistently to the items in the SF-8, as demonstrated by the very good internal consistency reliability. The item-total correlation for VT was relatively low, but acceptable. This was reasonable as the EFA results raised the possibility that VT belonging to a third domain, rather than the physical and mental health domains. Regarding the sub-scale scores, the floor effects (i.e., the percentage of participants with the lowest sub-scale score or worse health status) were very small. Although the ceiling effects (i.e., the percentage of participants with the highest sub-scale score or best health status) were relatively large, our percentages were comparable to those reported in a previous study on SF-12 using a Chinese population [26]. The SF-8 was designed to measure the impact of health problems on HRQOL. Therefore, it was not surprising that we observed larger ceiling effects in a general population sample. Overall, the Chinese version SF-8 was able to capture the range of health status in the urban Chinese population.

Our study is the first to confirm that the 2-factor structure (physical and mental health) of the SF-36 and SF-8 found in the US is reasonable for the Chinese version SF-8 among city residents of China. Therefore, the US norm-based scoring algorithm, developed assuming the 2-factor model, could be used for calculating the PCS and MCS summary scores among Chinese. Our CFA results do not imply that the US norm-based scoring algorithm is the best scoring method to reproduce the summary scores among Chinese. However, using the algorithm allows comparisons of HRQOL across different populations and countries, which is desired by many [27]. On the other hand, prior studies have reported notable differences between the US norm-based weights and country specific sample-based weights for the 2-factor model, potentially due to cultural differences in health perceptions [5, 28]. Thus, the PCS and MCS summary scores should be interpreted with caution. Individual sub-scale scores need to be considered with the summary scores.

Our results showed that a 3-factor model (physical, mental, and overall health) had slightly better fit of the data than the traditional 2-factor model. This is consistent with the results from Wang et al., in which the authors also found a 3-factor model with GH and VT loading on a separate third domain. Although few studies are available on the factor structure of SF-8, numerous studies on the SF-36 have reported its factor structures being different in Asia, including China [29, 30], Singapore [24], Taiwan [31], and Japan [32], compared to the US and Europe. The differences in factor structure across countries have led to the support for 3-factor models of the SF-36. Keller et al. proposed a third “general well-being” factor as an addition to the traditional “physical” and “mental” health factors based on data from the US and Europe [15]. Buchcik et al. also suggested that “HRQoL is influenced by more than a Mental and a Physical Component” and “a third component (e.g. general well-being) should be included” [33]. In Asia, Huang et al. found that a model with 3 s-order factors (“physical”, “mental”, and “social”) and 1 third-order factor (“health”) best fit the SF-36 data from the general Taiwan population [34]. Therefore, it is not surprising that our findings provide preliminary support for a 3-factor structure of the SF-8 among the Chinese population. However, more future studies are needed to further explore the factor structure of the SF-8.

Our study has several limitations. First, this study was cross-sectional and did not allow repeated measurements. Therefore, the SF-8 was only administered on a single occasion. We were unable to evaluate some of the potentially important psychometric properties such as test-retest reliability or sensitivity to change. Second, limited by the length of our survey, data on chronic conditions were not collected. Thus, we did not evaluate the usefulness of the SF-8 in discriminating among individuals with different levels of chronic conditions. Future studies are needed to further analyze the psychometric properties of the SF-8 Chinese version, including the test-retest reliability, using a large sample. Third, since our data were collected with RDD telephone surveys, this study has the limitations of any RDD study, such as selection bias. Fourth, there is no published data on the demographic characteristics of our target population, adults who live in the cities, which has prevented us from comparing these characteristics between our sample and the target population. It is therefore difficult to evaluate the representativeness of our sample. However, given the rigorous study design (i.e., multi-stage stratified sampling scheme) and data collection process (i.e., CATI conducted in a survey center), it is reasonable to assume that our results are generalizable to all city residents in China.

## Conclusions

To our knowledge, this study is the first to confirm the traditional 2-factor structure of SF-8 (PCS and MCS) using a large, representative sample from China. We have shown that the SF-8 Chinese version is feasible, reliable, and valid. Our findings support the use of the SF-8 summary scores for assessing general HRQOL among Chinese. More future studies are needed to evaluate the validity of a 3-factor structure for the SF-8 among the Chinese population.

## Abbreviations

BP: Bodily pain
CATI: Computer-assisted telephone interviews
CFA: Confirmatory factor analysis
CFI: Comparative fit index
EFA: Exploratory factor analysis
GH: General health perceptions
HRQOL: Health-related quality of life
IQOLA: International Quality of Life Assessment
MCS: Mental component score
PCS: Physical component score
PF: Physical functioning
RE: Role limitations due to emotional problems
RMSEA: Root mean square error of approximation
RP: Role limitations due to physical health problems
SF: Social functioning
SF-36: Short Form-36
SF-8: Short Form-8
SRMR: Standardized root mean square residual
VT: Vitality
